# Breeding Sites of Bluetongue Virus Vectors, Belgium

**DOI:** 10.3201/eid1603.091311

**Published:** 2010-03

**Authors:** Jean-Yves Zimmer, Claude Saegerman, Bertrand Losson, Eric Haubruge

**Affiliations:** University of Liege, Liege, Belgium

**Keywords:** Culicoides, breeding sites, ecology, vector, bluetongue, bluetongue virus, viruses, cowshed, Belgium, letter

**To the Editor:** Bluetongue (BT) is an emerging disease of ruminants in northern Europe (*1**,**2*). This disease was reported in August 2006 in the Netherlands and a few days later in Belgium. In 2006, animals in the Netherlands, Belgium, and Germany were affected. In contrast to 2006, when BT virus (BTV) was identified in ≈2,000 enclosures on farms, BTV was identified in >40,000 farm buildings containing ruminants in 2007; many infected animals had severe disease. In addition, the virus expanded its range to include large areas of France, Denmark, the United Kingdom, Switzerland, and the Czech Republic (*2*).

In 2008, BTV serotype 8 (BTV-8) continued its spread across Europe and showed virulence in France where 26,925 BTV-8 outbreaks were reported (*3*). This observation indicates possible overwintering of the vector from year to year. However, the mechanism of overwintering is not clear. The biting midges responsible for transmission of BTV in northern Europe belong to the genus *Culicoides*, but only few species are vectors of this virus (*2*).

During the winter of 2006–2007, Losson et al. (*1*) monitored the presence of biting midges inside farm buildings. Zimmer et al. (*4*) observed potential vectors of BTV inside a sheepfold during the winter of 2007–2008 and in farm buildings in 2008. These authors suggested that *Culicoides* spp. may be more abundant indoors than outdoors when animals are kept in these buildings. Breeding sites of bluetongue vector species have been found near farms (silage residues) (*5*) and in neighboring meadows (overwintering cattle dung and silt along a pond) (*5**,**6*) but not inside sheds.

We conducted a study on 5 cattle farms in Belgium during February–October 2008. Three samplings were performed: the first in late February, the second in mid-June, and the third in late October. Soil samples (15 biotopes) were collected inside cowsheds. These samples were incubated at 24°C to enable adult midges to emerge. All *Culicoides* specimens were identified by sex and to the species level by using the morphologic key of Delécolle (*7*).

Among 15 soil biotopes obtained from farm buildings, only 1 showed the emergence of adult *Culicoides* biting midges. At a cattle farm in Spy (50°28′31′′N, 4°40′39′′E), we found that dried dung adhering to walls inside animal enclosures and used animal litter was a breeding site for the *C*. *obsoletus/scoticus* complex (Table). Only 25% of emerging *Culicoides* midges were females.

**Table:** *Culicoides* species obtained from dried dung samples inside a cowshed, Spy, Belgium, 2008

Sampling period	Carbon: nitrogen index	No. *Culicoides* specimens	*Culicoides* species	
			*C. obsoletus* males	*C. obsoletus/scoticus* females
Late February	19.5	53	40	13
Mid-June	12.8	13	10	3
Late October	12.5	3	2	1

We observed that *C*. *obsoletus/scoticus* complex midges are more prevalent in soil samples with a high carbon:nitrogen (C:N) index; this index indicates the amount of organic matter in soil. C:N indices between 15 and 30 support production of humus and ensure good microbial growth. In addition, larvae of *Culicoides* spp. feed on organic material and microorganisms in soil (*8*).

Our observations suggest that biting midges can complete their life cycle in animal enclosures. This finding is consistent with the high capture rates of nulliparous (empty and unpigmented abdomens) (*9*) adult midges observed when suction light traps (Onderstepoort Veterinary Institute, Onderstepoort, South Africa) were used on cattle farms during April–May 2007 (*4*).

We identified a breeding site for the primary BTV vector in a cowshed in northern Europe (*10*). Vectors feed on blood, overwinter inside cowsheds (*1*), lay eggs, and larvae develop under such conditions. These observations could explain the persistence of BTV from year to year despite fairly harsh winters.

Hygienic measures on farms could reduce midge populations and improve efficacy of vaccination campaigns against BT in Europe. We strongly recommend that such integrated control strategies be evaluated. Removal of residual animal feed and feces on farms and of material from silage structures and sheds, particularly deposits of manure adhering to walls of sheds and used litter, are simple and inexpensive measures that should be implemented. However, their success will depend on active participation by farmers.
